# The major genetic risk factor for severe COVID-19 does not show any association among South Asian populations

**DOI:** 10.1038/s41598-021-91711-4

**Published:** 2021-06-11

**Authors:** Prajjval Pratap Singh, Anshika Srivastava, Gazi Nurun Nahar Sultana, Nargis Khanam, Abhishek Pathak, Prashanth Suravajhala, Royana Singh, Pankaj Shrivastava, George van Driem, Kumarasamy Thangaraj, Gyaneshwer Chaubey

**Affiliations:** 1 Cytogenetics Laboratory, Department of Zoology, Banaras Hindu University, Varanasi, Uttar Pradesh 221005 India; 2 Centre for Advanced Research in Sciences (CARS), Genetic Engineering and Biotechnology Research Laboratory, University of Dhaka, Dhaka, 1000 Bangladesh; 3 Department of Neurology, Institute of Medical Sciences, Banaras Hindu University, Varanasi, India; 4 Department of Biotechnology and Bioinformatics, Birla Institute of Scientific Research Statue Circle, Jaipur, Rajasthan India; 5 Department of Anatomy, Institute of Science, Banaras Hindu University, Varanasi, Uttar Pradesh 221005 India; 6 Department of Home (Police), DNA Fingerprinting Unit, State Forensic Science Laboratory, Government of MP, Sagar, India; 7 Institut Für Sprachwissenschaft, Universität Bern, Länggassstrasse 49, 3012 Bern, Switzerland; 8 CSIR-Centre for Cellular and Molecular Biology, Hyderabad, India; 9 Centre for DNA Fingerprinting and Diagnostics, Hyderabad, India

**Keywords:** Evolutionary genetics, Molecular evolution, Population genetics, Risk factors

## Abstract

With the growing evidence on the variable human susceptibility against COVID-19, it is evident that some genetic loci modulate the severity of the infection. Recent studies have identified several loci associated with greater severity. More recently, a study has identified a 50 kb genomic segment introgressed from Neanderthal adding a risk for COVID-19, and this genomic segment is present among 16% and 50% people of European and South Asian descent, respectively. Our studies on *ACE2* identified a haplotype present among 20% and 60% of European and South Asian populations, respectively, which appears to be responsible for the low case fatality rate among South Asian populations. This result was also consistent with the real-time infection rate and case fatality rate among various states of India. We readdressed this issue using both of the contrasting datasets and compared them with the real-time infection rates and case fatality rate in India. We found that the polymorphism present in the 50 kb introgressed genomic segment (rs10490770) did not show any significant correlation with the infection and case fatality rate in India.

## Introduction

Since the beginning of COVID-19 pandemic, it has been observed that people with a different ethnic background and country or continent of origin have variable degrees of susceptibility^1,2^. Though there are a few well known factors for higher susceptibility, e.g. age and comorbidity^3,4^, the hospitalisation of younger healthy people has also been reported^5^. Recent genome wide association study has identified a gene cluster at chromosome 3 as well as the ABO gene at chromosome 9 associated with the severe risk factor for COVID-19 among Europeans^6^. Subsequently, the COVID-19 Host Genetics Initiative has corroborated this result^7^. The worldwide meta-analysis of the COVID-19 Host Genetics Initiative has identified 13 genetic loci associated with higher susceptibility or higher severity^8^.

Zeberg and Pääbo^9^ have identified a risk haplotype of 50 kb size introgressed from Neanderthals, which they called the ‘Neanderthal core haplotype’. This risk haplotype was found to be present with an allele frequency of 30% among South Asians, 8% in Europeans and 4% among admixed Americans. The peak carrier frequency was estimated among the Bangladeshi population, where 63% carried at least one copy of this haplotype. The study also cited twice the risk of mortality among people of Bangladeshi extraction living in the UK as opposed to the native population of Brittanic pedigree^10^.

Conversely, three of our studies on *ACE2*, the gateway of SARS-CoV-2, identified a haplotype, shared among South Asians and East Eurasians, likely protecting them from severe risk^11–13^. Additionally, the spatial distribution of this haplotype showed strong association with the low infection as well as low case fatality rate (CFR)^13^. To resolve this discrepancy between the two sets of findings and the associated claims, we have extracted a SNP (rs10490770) reported to be associated with the high risk for COVID-19^9^, from our published and unpublished genome wide datasets (Supplementary Table S1), and looked for existing association with the state-wise COVID-19 data of India.

## Materials and methods

Zeberg and Pääbo^9^ have mainly discussed about the SNP rs35044562. However, they reported 12 other SNPs present in the ‘Neanderthal core haplotype’ that are in high linkage disequilibrium (r^2^ > 0.98) (Supplementary Table S2). SNP rs10490770 showed high LD (r^2^ = 0.99) with the SNP rs35044562. The genome-wide genotype data by Illumina tagged rs2285666 and rs10490770 SNPs in their panel. Therefore, we searched the genotype datasets generated by this platform. The frequency data for both of the SNPs from various Indian populations were extracted using Plink 1.9^14^, from 1000 genome project data phase 3^15^, data published by the Estonian Biocentre^16–19^ and our newly genotyped samples for various Indian states and Bangladesh (Supplementary Table S1). In addition to our previous study^13^, more samples were added for the SNP rs2285666. The state-wise COVID-19 infection and CFR datasets were extracted from https://www.covid19india.org/. The regression estimations and plots were built by https://www.graphpad.com/quickcalcs/linear1/ and reverified by the Microsoft Excel regression calculations. We have also used Pearson’s correlation coefficient test^20^ to evaluate the effect of both the SNPs. The spatial distribution of both SNPs were drawn using the web tool available at https://www.datawrapper.de/.

## Results and discussion

**Figure 1:**
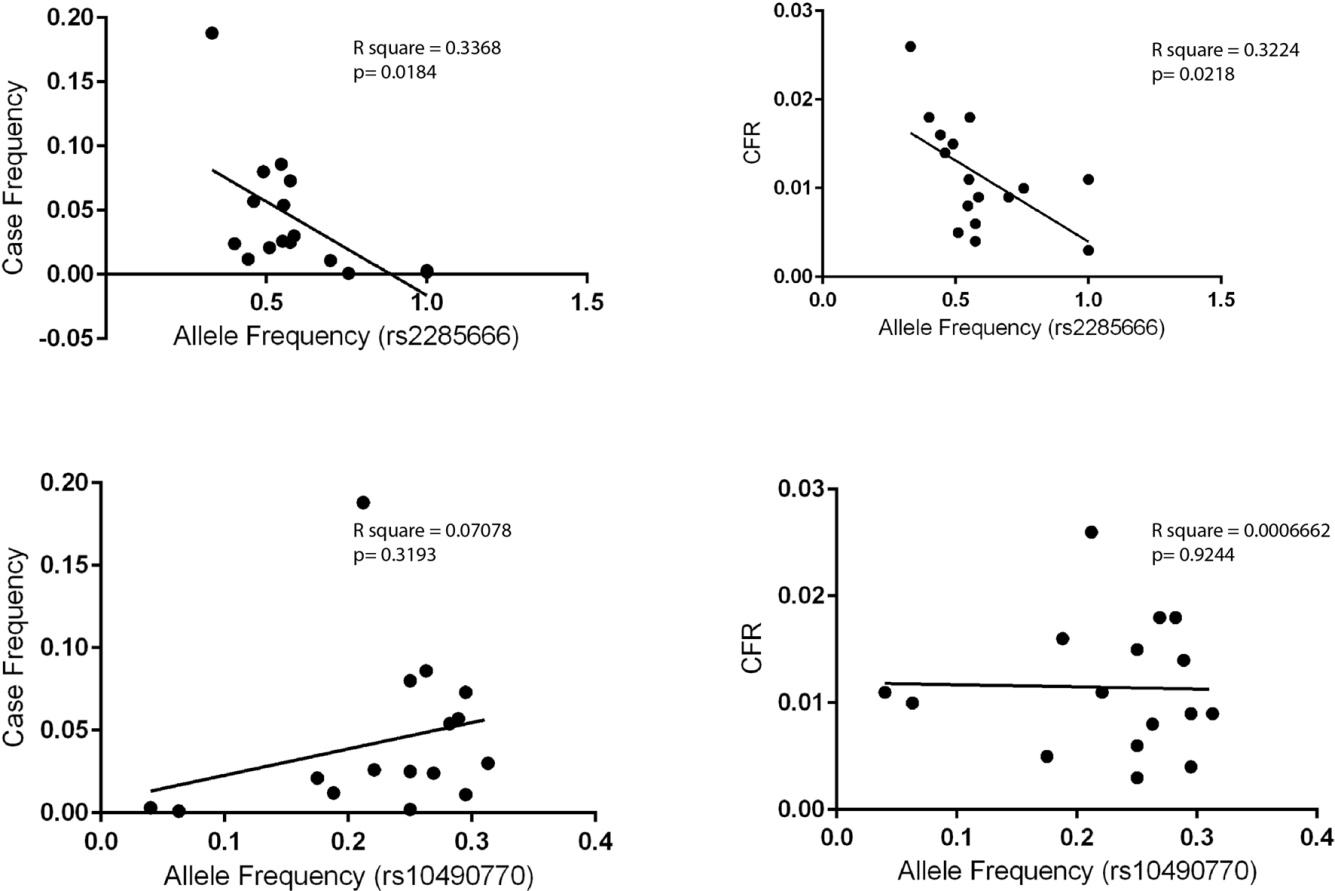
The regression analyses of rs2285666 (*ACE2*) and rs10490770 (*LZTFL1*) with the COVID-19 cases as well as case fatality rate in various states of India (Supplementary Table S1).

In contrast to the conclusions drawn by Zeberg and Pääbo^9^, our study on *ACE2* identified a haplotype that is frequent among South Asians and East Eurasians^11–13^. This haplotype is derived by a polymorphism rs2285666 responsible for elevated expression of *ACE2*. We have found high inverse correlation of this haplotype with the state-wise cases as well as the case-fatality rate (CFR) among Indian populations^13^. This correlation was significant at various timelines of the pandemic in India (Table 1). We verified the statistical tests with the updated data up to December 2020 and found these data to be consistent with previous observations (Fig. 1 and Supplementary Fig. S1). Thus, it is likely that the *ACE2* SNP rs2285666 has played a significant role in modulating the susceptibility to the disease among Indian populations.

**Table 1:** Estimates of Pearson correlation coefficient for the rs2285666 and rs10490770 with the real-time COVID-19 cases as well as case fatality rate among Indian populations. The significant values are shown in bold letters.

		rs2285666 (T)	rs10490770 (C)	FreqMay2020	FreqAug2020	FreqDec2020	CFRAug2020	CFRAug2020
rs2285666	Pearson correlation Sig. (2-tailed)	1	− 0.428 0.098	− 0.588* 0.017	− 0.558* 0.025	− 0.580* 0.018	− 0.649** 0.007	**− 0.568** 0.022
rs10490770	Pearson correlation Sig. (2-tailed)	− 0.428 0.098	1	0.204 0.450	0.164 0.543	0.266 0.319	0.244 0.362	− 0.026 0.924

*Correlation is significant at the 0.05 level (2-tailed).

**Correlation is significant at the 0.01 level (2-tailed).

In our search of the SNPs reported to be associated with high risk by Zeberg and Pääbo^9^, we found rs10490770 from genome-wide datasets^17,18,21–23^. We applied the same tests done for the *ACE2* SNPs (Fig. 1). The state-wise frequency variation of this SNP did not show any association either with the number of cases or the CFR (Table 1 and Supplementary Fig. S1). We repeated these regression tests for the number of cases as well as the CFR data, obtained during all the three months. However, none of them showed any association with the rs10490770 (p > 0.3) (Table 1). It is interesting to note that this SNP (rs10490770) has been found to be associated with disease severity in the global data^8^. However, the lack of association for rs10490770 with COVID-19 cases or CFR in India is striking and suggests instead a complex susceptibility response among Indian populations. Along with the complex genetic structure^11,24^, socio-economic status^25^ and hygiene^26^ may have contributed to such a complex scenario. Furthermore, a detailed clinical and genome-wide association study on Indian COVID-19 patients would be useful to resolve this complexity.

Zeberg and Pääbo^9^ used the data of higher susceptibility to the disease among the Bangladeshi population living in UK^10^ to support their findings. By considering the effect of sex, age, socio-economic deprivation and region, this report found that people of Bangladeshi origin had double the risk of mortality as compared to people of British origin. However, the higher mortality rate for Bangladeshi population in the UK needs more detailed investigation on comorbidity, genetic admixture as well as local environment and socio-economic circumstances in their particular British context. More importantly, a similar trend had also been observed among admixed Americans, where some of the same qualifications may apply *mutatis mutandis*^27–29^. Furthermore, it is notable that among the Bangladeshi samples analysed by us, the tribal populations of Bangladesh showed almost three times less frequency of rs10490770 (Supplementary Table S1). This is likely due to the different population histories of the caste and tribal populations of Bangladesh^30,31^. The Tibeto-Burman speakers of Bangladesh show a closer genetic affinity with the East and Southeast Asian populations, whereas the Indo-European speaking caste populations incline with the Indian populations. Therefore, it is advised explicitly to differentiate between the caste and tribal populations while making any statement about Bangladeshi populations. Significantly, our data also show that the incidence of the allele rs2285666 has been found to occur in the highest frequency of 100% in Indian populations such as the Nishi and Kokborok (Tripuri), who represent Trans-Himalayan language communities (Supplementary Fig. S1 and Supplementary Table S1). As a linguistic phylum, the Trans-Himalayan language family is widespread in parts of eastern Eurasia and includes languages such as Tibetan, Burmese, Mandarin, Cantonese and Hokkien.

Thus, our extensive analyses on real-time data did not show any association of rs10490770 with the state-wise infection rates as well as CFRs, suggesting that the risk allele for COVID-19 in Europe does not play a significant role in COVID-19 severity in South Asia.

## Supplementary Information

Supplementary Information.

## Data Availability

All datasets generated for this study are included in the article/Supplementary Material.
