# Publisher Correction: The major genetic risk factor for severe COVID‑19 does not show any association among South Asian populations

**DOI:** 10.1038/s41598-021-94864-4

**Published:** 2021-08-05

**Authors:** Prajjval Pratap Singh, Anshika Srivastava, Gazi Nurun Nahar Sultana, Nargis Khanam, Abhishek Pathak, Prashanth Suravajhala, Royana Singh, Pankaj Shrivastava, George van Driem, Kumarasamy Thangaraj, Gyaneshwer Chaubey

**Affiliations:** 1 Cytogenetics Laboratory, Department of Zoology, Banaras Hindu University, Varanasi, Uttar Pradesh 221005 India; 2 Centre for Advanced Research in Sciences (CARS), Genetic Engineering and Biotechnology Research Laboratory, University of Dhaka, Dhaka, 1000 Bangladesh; 3 Department of Neurology, Institute of Medical Sciences, Banaras Hindu University, Varanasi, India; 4 Department of Biotechnology and Bioinformatics, Birla Institute of Scientific Research Statue Circle, Jaipur, Rajasthan India; 5 Department of Anatomy, Institute of Science, Banaras Hindu University, Varanasi, Uttar Pradesh 221005 India; 6 Department of Home (Police), DNA Fingerprinting Unit, State Forensic Science Laboratory, Government of MP, Sagar, India; 7 Institut Für Sprachwissenschaft, Universität Bern, Länggassstrasse 49, 3012 Bern, Switzerland; 8 CSIR-Centre for Cellular and Molecular Biology, Hyderabad, India; 9 Centre for DNA Fingerprinting and Diagnostics, Hyderabad, India

Correction to: *Scientific Reports* 10.1038/s41598-021-91711-4, published online 11 June 2021

In the original version of this Article, Kumarasamy Thangaraj was omitted as a corresponding author. Correspondence and request for materials should also be addressed to thangs@ccmb.res.in. In addition, the email address of the co-corresponding author Gyaneshwer Chaubey was incorrectly given as thangs@ccmb.res.in. Correspondence and request for materials should also be addressed to gyaneshwer.chaubey@bhu.ac.in.

The original Article has been corrected.

