# Supplementary material for: The major genetic risk factor for severe COVID-19 does not show any association among South Asian populations
Source: Sci Rep. 2021 Jun 11;11:12346. doi: 10.1038/s41598-021-91711-4 (PMC8196069; doi:10.1038/s41598-021-91711-4)
Supplement: Supplementary file 1 — Supplementary Information. [file 41598_2021_91711_MOESM1_ESM.docx]

**Supplementary Figure 1.** The state-wise frequency distribution of both of the compared SNPs in our analysis.


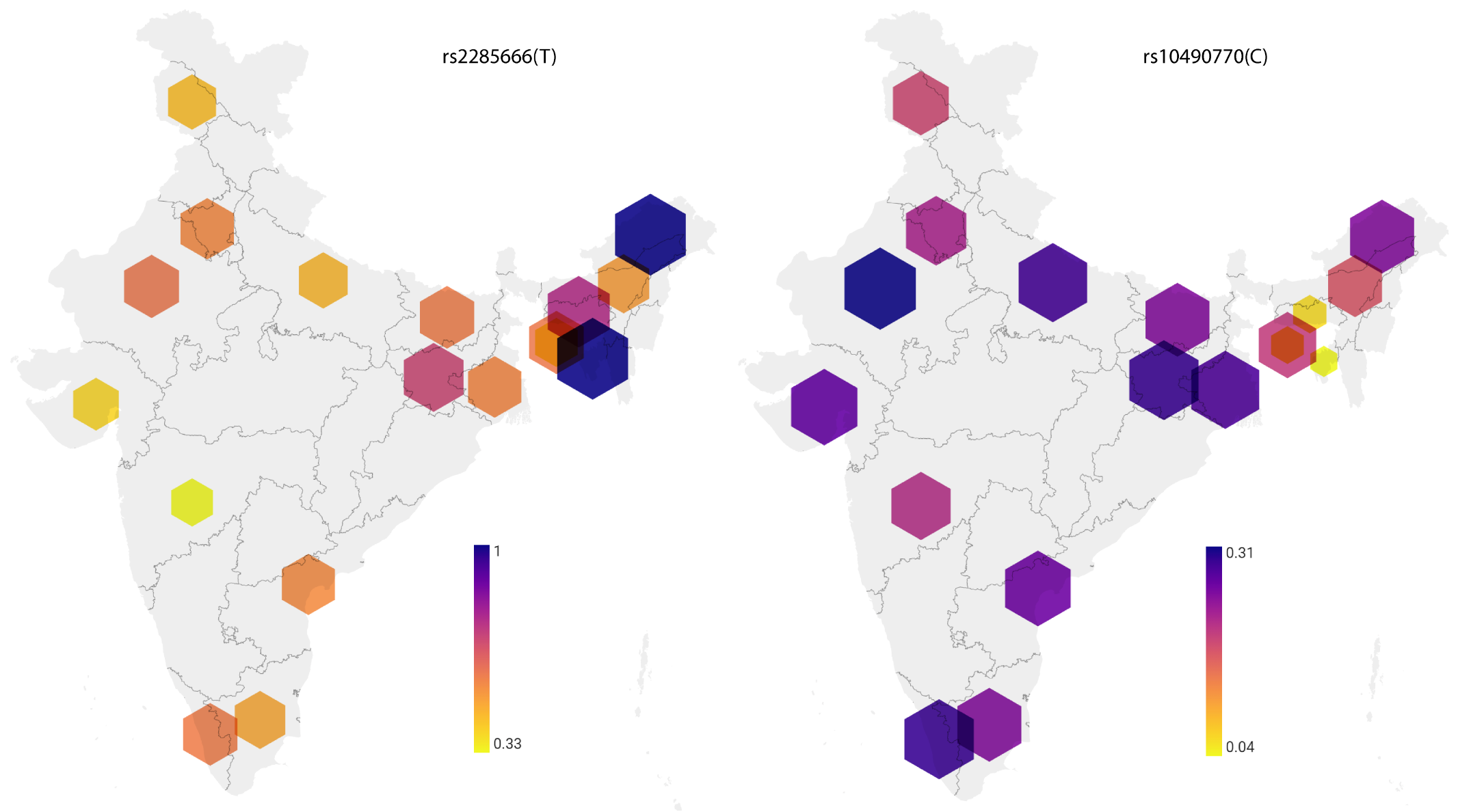


**Supplementary Table 1.** The Indian state-wise frequency of both of the SNPs used in the present study. The frequency of cases as well as case-fatality rates at various timeline of this study have been shown.

**Supplementary Table 2.** The details of COVID-19 susceptibility associated SNPs observed in various studies.

**References**

1000 Genomes Project Consortium *et al.* A map of human genome variation from population-scale sequencing. *Nature* **467**, 1061–73 (2010).

Chaubey, G. *et al.* “Like sugar in milk”: reconstructing the genetic history of the Parsi population. *Genome Biol.* **18**, 110 (2017).

Ganna, A. Mapping the human genetic architecture of COVID-19 by worldwide meta-analysis. *medRxiv* 2021.03.10.21252820 (2021) doi:10.1101/2021.03.10.21252820.

Pathak, A. K. *et al.* The Genetic Ancestry of Modern Indus Valley Populations from Northwest India. *Am. J. Hum. Genet.* **103**, 918–929 (2018).

Srivastava, A. *et al.* Genetic Association of ACE2 rs2285666 Polymorphism With COVID-19 Spatial Distribution in India. *Front. Genet.* **11**, 1163 (2020).

Tätte, K. *et al.* The genetic legacy of continental scale admixture in Indian Austroasiatic speakers. *Sci. Rep.* **9**, 3818 (2019).

Zeberg, H. & Pääbo, S. The major genetic risk factor for severe COVID-19 is inherited from Neanderthals. *Nature* 1–3 (2020).
